# Proprotein convertase subtilisin/kexin type 9 deficiency in extrahepatic tissues: emerging considerations

**DOI:** 10.3389/fphar.2024.1413123

**Published:** 2024-07-30

**Authors:** Fengyuan Lu, En Li, Xiaoyu Yang

**Affiliations:** ^1^ The Second Affiliated Hospital, Zhengzhou University, Zhengzhou, China; ^2^ School of Basic Medical Sciences, Zhengzhou University, Zhengzhou, China

**Keywords:** PCSK9, PCSK9 deficiency, PCSK9 inhibition, PCSK9 monoclonal antibody, low-density lipoprotein receptors

## Abstract

Proprotein convertase subtilisin/kexin type 9 (PCSK9) is primarily secreted by hepatocytes. PCSK9 is critical in liver low-density lipoprotein receptors (LDLRs) metabolism. In addition to its hepatocellular presence, PCSK9 has also been detected in cardiac, cerebral, islet, renal, adipose, and other tissues. Once perceived primarily as a “harmful factor,” PCSK9 has been a focal point for the targeted inhibition of both systemic circulation and localized tissues to treat diseases. However, PCSK9 also contributes to the maintenance of normal physiological functions in numerous extrahepatic tissues, encompassing both LDLR-dependent and -independent pathways. Consequently, PCSK9 deficiency may harm extrahepatic tissues in close association with several pathophysiological processes, such as lipid accumulation, mitochondrial impairment, insulin resistance, and abnormal neural differentiation. This review encapsulates the beneficial effects of PCSK9 on the physiological processes and potential disorders arising from PCSK9 deficiency in extrahepatic tissues. This review also provides a comprehensive analysis of the disparities between experimental and clinical research findings regarding the potential harm associated with PCSK9 deficiency. The aim is to improve the current understanding of the diverse effects of PCSK9 inhibition.

## 1 Introduction

Proprotein convertase subtilisin/kexin type 9 (PCSK9) was originally termed neural apoptosis-regulated convertase 1 because of its robust expression in the telencephalons of embryonic mice (Seidah et al., 2003). The expression of PCSK9 correlates with neural progenitor cell differentiation into more abundant neuronal lineages (Seidah et al., 2003). Subsequently identified as the third pathogenic gene associated with familial hypercholesterolemia, along with low-density lipoprotein receptors (LDLRs) and apolipoprotein B (ApoB) genes, PCSK9 has been extensively studied concerning its interplay with lipid homeostasis (Abifadel et al., 2003). The modulation of lipid levels by PCSK9 occurs mainly through the downregulation of LDLRs on the hepatocyte membrane surface, which inhibits the degradation of low-density lipoprotein cholesterol (LDL-C) in circulation (Rudenko et al., 2002; Lagace et al., 2006). Mechanistically, PCSK9 lacks proteolytic activity. Instead, it binds to the epidermal growth factor fragment of LDLR through its catalytic domain, facilitating delivery of LDLR to the endosome-lysosome for degradation. For example, when Gypenoside LVI is used to inhibit the expression of PCSK9 in HepG2 cells, an increase in the density of LDLR on the HepG2 cell membrane can be detected, along with an observed increase in red fluorescently labeled LDL in the cytoplasm (Wang et al., 2021). Subsequently, PCSK9 re-circulates to the cellular outer membrane to initiate further LDLR interactions (Bottomley et al., 2009; Cui et al., 2015). PCSK9 also orchestrates the degradation of membrane receptors, such as low-density lipoprotein receptor-related protein 1 (LRP1/ApoER) (Fu et al., 2017), low-density lipoprotein receptor-related protein 8 (LRP8/ApoER2) (Poirier et al., 2008), cluster of differentiation 36 (CD36) (Demers et al., 2015), cluster of differentiation 81 (CD81) (Le et al., 2015), very-low-density lipoprotein receptor (VLDLR) (Poirier et al., 2008), and epithelial sodium channel (ENaC) (Sharotri et al., 2012) through a similar pathway. The role of PCSK9 as a predictor of the risk of atherosclerosis is evident from its gain-of-function mutation, which correlates with the occurrence of conditions, such as coronary heart disease (CAD), abdominal aortic aneurysm, peripheral artery disease, and stroke (Ferreira et al., 2020; Qin et al., 2021; Sawada et al., 2022). In contrast, individuals with loss-of-function (LOF) mutations in the PCSK9 gene have lower serum LDL-C levels, which reduces the risk of coronary heart disease and stroke (Kent et al., 2017). These insights substantiate the potential of PCSK9 inhibition as a strategy to lower LDL-C levels. Currently, PCSK9 monoclonal antibodies are the most extensively employed inhibitors. These antibodies reduce serum LDL-C levels by 60%–70% and deliver sustained benefits to individuals with established CAD (ODonoghue et al., 2022).

**FIGURE 1:**
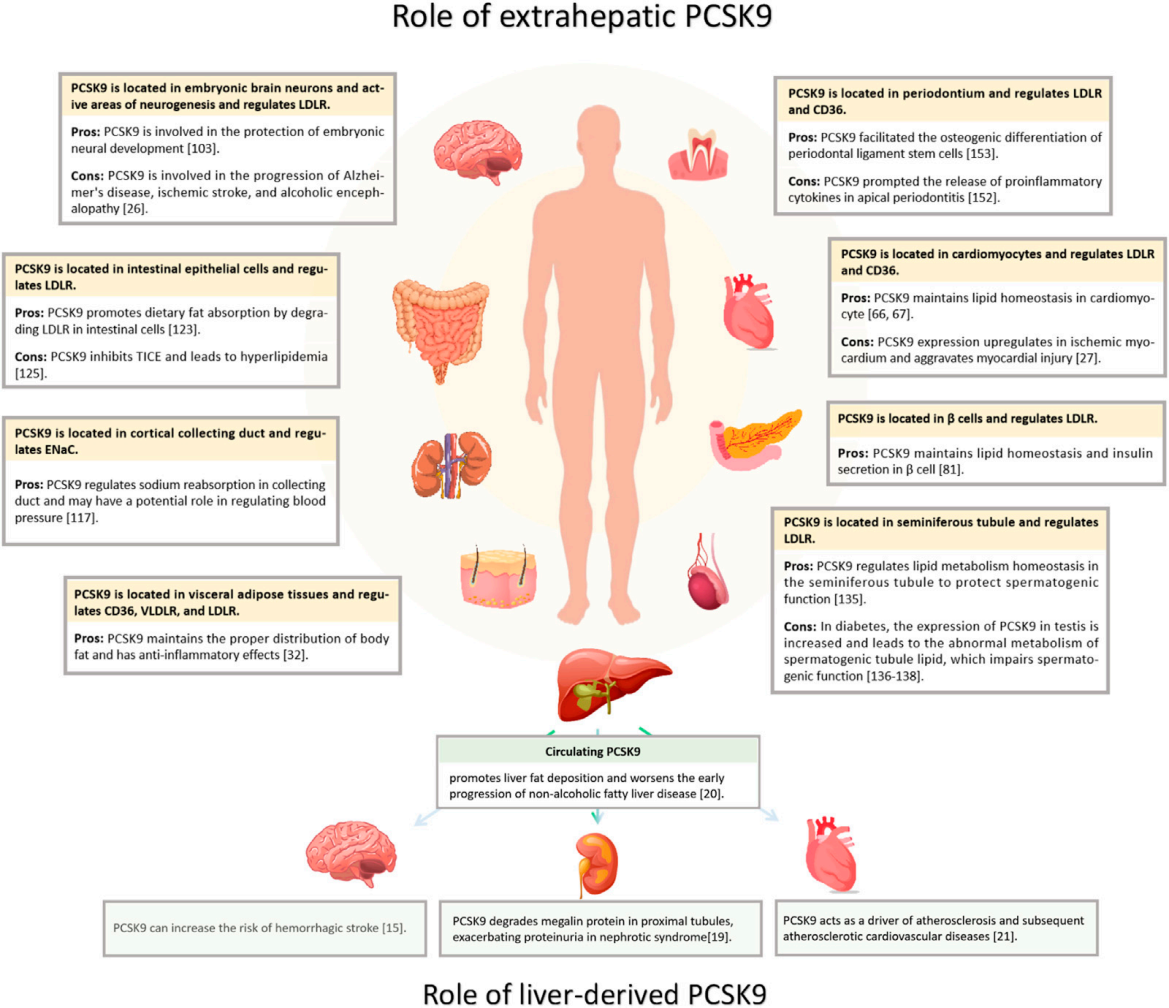
Distribution of PCSK9 expression and effects. PCSK9 is located in various extrahepatic organs, including heart, brain, islets, kidney, intestine, testis, and periodontium, and in adipose tissue. The yellow segments indicate PCSK9 locations; Pros, the protective impact of PCSK9; Cons, detrimental effects.

Hepatic PCSK9 is abundantly expressed and is the primary source of serum PCSK9 (Zaid et al., 2008). Extrahepatic tissues, such as the heart, brain, intestine, kidney, and pancreas, also secrete PCSK9 (Figure 1) (Qin et al., 2021; Momtazi-Borojeni et al., 2022; Boutari et al., 2023; Skeby et al., 2023). PCSK9 operates in an autocrine manner in these tissues and does not constitute circulating PCSK9 (Levy et al., 2013; Barisione et al., 2021; Lin et al., 2021). In the heart, PCSK9 acts as an inflammatory mediator expressed in ischemic myocardial cells, fostering local inflammation and cell death (Wang et al., 2020). In the brain, PCSK9 binds to LRP1 and impedes β-amyloid protein clearance, and interacts with various inflammatory factors, underpinning neurodegenerative conditions like Alzheimer’s disease (AD) (Mazura et al., 2022). Therefore, in addition to affecting LDL-C levels, PCSK9 has several other functions, and its inhibition offers a novel therapeutic avenue for extrahepatic organ diseases, such as acute myocardial infarction (AMI) and AD (Abuelezz and Hendawy, 2021).

Although studies have focused on targeted PCSK9 inhibition for disease treatment, it is crucial to acknowledge its role in maintaining normal physiological functions in multiple tissues (Şener and Tokgözoğlu, 2023). The inhibition of PCSK9 increases various lipoprotein receptors, such as LDLR, VLDLR, and CD36, significantly enhancing the ability of cells to absorb lipids (Poirier et al., 2008; Bottomley et al., 2009; Cui et al., 2015; Demers et al., 2015). Unlike the liver, many extrahepatic tissues struggle to manage excessive lipid uptake by redirecting excess cholesterol into the liver through *high-density lipoprotein* packaging (Lewis and Rader, 2005). Thus, PCSK9 deficiency disrupts lipid homeostasis in extrahepatic cells by fostering excessive cholesterol uptake over metabolism, impairing damage to cells (Paul et al., 2016), while also contributing to various physiological activities, including brain nerve development, renal blood pressure regulation, and body fat distribution (Poirier et al., 2006; Baragetti et al., 2017).

This review summarizes the pleiotropic biological functions of PCSK9 and the potential physiological consequences of its deficiency, offering insights into the rationale for the widespread use of PCSK9 inhibitors.

## 2 PCSK9 gene transcriptional regulation

*In vitro*, both sterol regulatory element-binding protein 1-c (SREBP1-c) and sterol regulatory element-binding protein 2 (SREBP2) bind to the sterol regulator element (SRE) within the PCSK9 gene promoter, leading to the upregulation of PCSK9 expression (Jeong et al., 2008). However, *in vivo*, the primary regulator of PCSK9 is SREBP2 (Jeong et al., 2008). The expression of SREBP2 can be induced by low sterol concentrations and statin usage (Eberlé et al., 2004; Davignon and Dubuc, 2009). Positioned upstream of the SRE is histone nuclear factor P, which enhances PCSK9 expression by facilitating the acetylation of the PCSK9 promoter histone H4. This process greatly intensifies the transcriptional activity of SREBP2 in PCSK9 (Li and Liu, 2012). As a cholesterol-sensitive transcription factor of PCSK9, E2F transcription factor 1 directly elevates its transcriptional activity or enhances PCSK9 expression by activating SREBP1-c under insulin stimulation (Denechaud et al., 2016; Lai et al., 2017).

The binding sequence for hepatocyte nuclear factor 1-α (HNF1-α), situated 28bp upstream of SRE, is also important in upregulating PCSK9 expression. Mutations in this sequence disrupt the SRE sequence promoter (Li et al., 2009). Forkhead box class O 3a (FoxO3a) acts as an inhibitory transcription factor for PCSK9 and is activated by epigallocatechin gallate derived from green tea. The expression of FoxO3a potentially competes with the action of HNF1-α, inhibiting its effect (Cui et al., 2020). Notably, in a patient with drug-resistant hypercholesterolemia, serum PCSK9 concentrations surged by 15-fold, coinciding with the detection of HNF4-α overexpression, indicating that HNF4-α might also play a role in PCSK9 regulation (Lau et al., 2020). An association between PCSK9 and HNF4-α was also observed in a rat model with partial fat resection (Dettlaff-Pokora et al., 2019).

**FIGURE 2:**
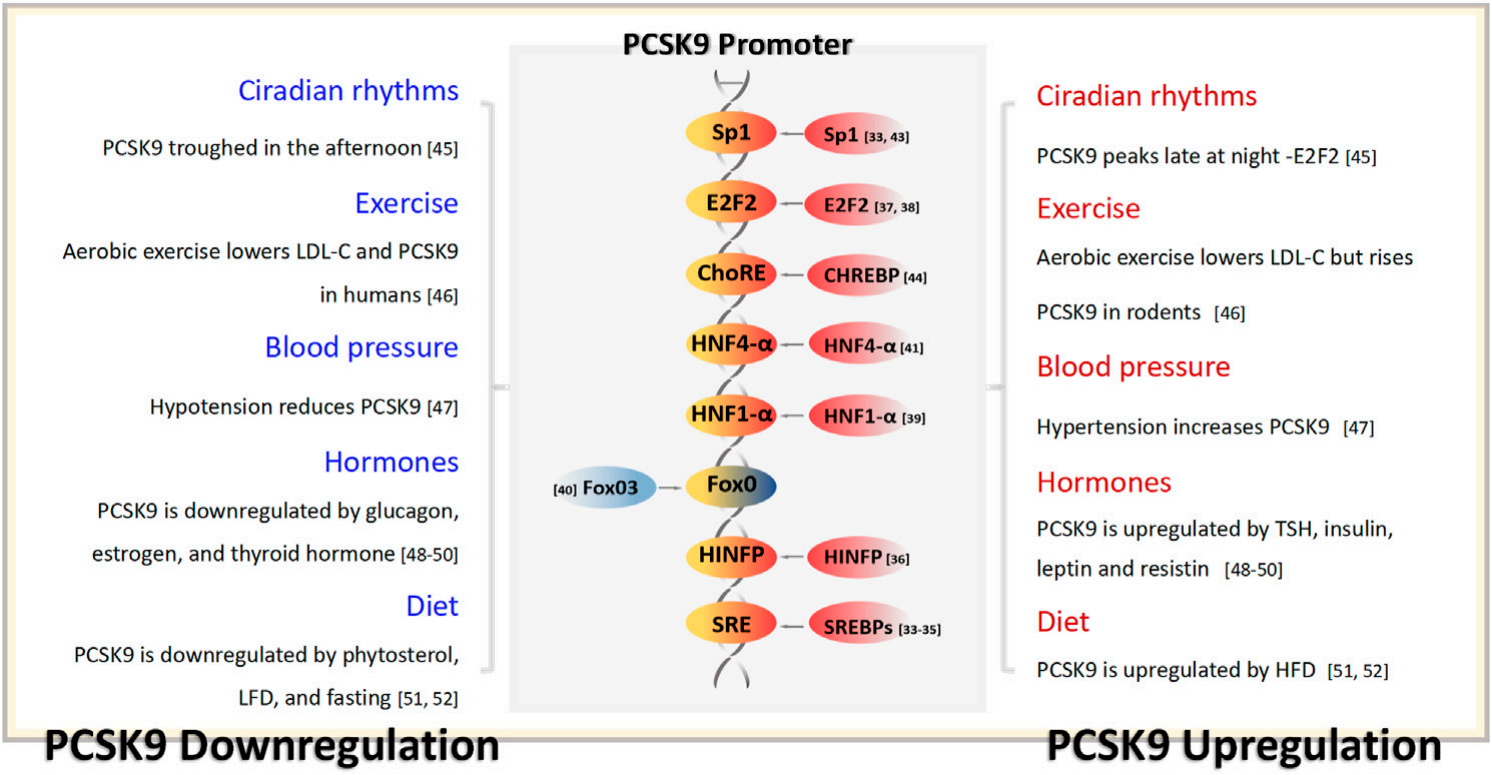
PCSK9 transcriptional regulation and the physiological factors capable of influencing the serum concentration of PCSK9. TSH, thyrotropin; LFD, low-fat diets; HFD, low-fat diets.

Further upstream of the SRE, the specificity protein 1 (sp1) binding site is believed to mediate PCSK9 transcription. Mutations at this site lead to significant changes in PCSK9 expression (Blesa et al., 2008; Jeong et al., 2008). The PCSK9 promoter region also features a binding site for carbohydrate-responsive element-binding protein (ChREBP). Metformin acts in a glucose-dependent manner and suppresses PCSK9 expression by inhibiting ChREBP (Hu et al., 2021). A comprehensive overview of the regulatory factors that influence PCSK9 expression is shown in Figure 2 (Costet et al., 2006; Cariou et al., 2010; Chen et al., 2014; Ooi et al., 2015; Levenson et al., 2017; Sponder et al., 2017; Guo WJ. et al., 2021; Sadik et al., 2022).

## 3 PCSK9 protects lipid metabolism in myocardium

### 3.1 Cardiomyocyte and lipid toxicity

Cardiomyocytes have substantial energy requirements and exhibit a distinct approach to energy metabolism. Remarkably, 60% of this energy is derived from fatty acid (FA) oxidation, primarily esterified FAs from circulation coupled with *de novo* FA synthesis (Razani et al., 2011). The precise composition of FAs is crucial, because they affect the physiological performance of the heart. Notably, FAs contribute to membrane phospholipids and cardiolipin, which are essential components of both cell and mitochondrial membranes (Chen et al., 2021). Additionally, certain entities, such as prostaglandin E2 (PGE2), PGD2, PGI2, linoleic acid, n-3 polyunsaturated FAs, and their metabolites exhibit cardiovascular safeguarding effects and improve ischemia-reperfusion injury (Moriyama et al., 2022). However, when the supply of FAs surpasses the capacity of β oxidation and storage as triacylglycerol, excessive accumulation of FAs leads to lipotoxicity (Lopaschuk et al., 2021). Clinically, lipotoxicity has been identified as a precursor of myocardial remodeling in cardiomyocytes of patients with diabetes, potentially driving ventricular remodeling and cardiac dysfunction (Ernande et al., 2010; Salvatore et al., 2021). Mechanistically, excessive intake of FAs by cardiomyocytes culminates in the accumulation of detrimental lipid metabolites, such as ceramide and diacylglycerol (DSouza et al., 2016). Concurrently, excessive FA intake disrupts the mitochondrial respiratory chain function and uncouples oxidative phosphorylation, impairing mitochondrial integrity and energy metabolism in cardiomyocytes (Goldberg et al., 2012).

### 3.2 PCSK9 deficiency in myocardium is associated with heart failure

Heart failure with preserved ejection fraction (HFpEF) is defined as heart failure with an ejection fraction ≥50%. HFpEF is frequently accompanied by metabolic risk factors that include type 2 diabetes mellitus (T2DM), obesity, and hypertension (Schiattarella et al., 2019). At a molecular level, these patients often exhibit cardiomyocyte lipid overload (Schiattarella et al., 2019). Excessive lipid uptake by cardiomyocytes is a pivotal factor in HFpEF (Leggat et al., 2021). Studies have revealed the significance of cardiomyocyte lipoprotein receptors (CD36, LDLR, and VLDLR) as conduits for the uptake and transport of FAs, which are strongly associated with progression of heart failure (Sung et al., 2011). In middle-aged wild type (WT) mice fed on a high-fat diet, increased CD36 expression reportedly causes cardiomyocyte hypertrophy (Sung et al., 2011). Patients with diabetes and HFpEF show elevated LDLR expression in the myocardium (Patel et al., 2020). In WT mouse models, VLDLR exacerbates the cardiomyocyte lipid burden and hastens the progression of heart failure (Perman et al., 2011).

Previous studies have confirmed that PCSK9 regulates the degradation of lipid uptake receptors, such as LDLR, CD36, and VLDLR (Poirier et al., 2008; Bottomley et al., 2009; Cui et al., 2015; Demers et al., 2015). Whether PCSK9 deficiency leads to enrichment of these receptors in the cardiomyocyte membrane, and also leads to HEpEF has garnered attention (Da Dalt et al., 2021). Da Dalt et al. first established that PCSK9 knockdown in mice resulted in HFpEF, as evidenced by increased left ventricular posterior wall thickness and reduced exercise capacity (Da Dalt et al., 2021). Cardiomyocytes from PCSK9 knockout mice exhibited elevated LDLR and CD36 levels, accompanied by substantial lipid droplet accumulation around the mitochondria (Da Dalt et al., 2021). The authors also described that, remarkably, liver-specific PCSK9 knockout mice displayed none of these changes, suggesting that myocardial autocrine PCSK9 plays a cardioprotective and lipid regulatory role (Da Dalt et al., 2021). This was further validated in a cardiomyocyte-specific PCSK9 knockout model by Laudette et al. (2023). Reduced PCSK9 expression in the myocardium resulted in signs of heart failure, left ventricular dilatation, myocardial interstitial fibrosis, and pulmonary congestion in middle-aged mice (28 weeks old), ultimately leading to mortality within 8 weeks (Laudette et al., 2023). The authors also described that *in vitro* cultured cardiomyocytes with silenced PCSK9 exhibited changes in mitochondrial membrane lipid components, increased levels of free FAs, decreased electron transfer chain activity, and mitochondrial distortion and breakage (Laudette et al., 2023).

Individuals harboring p.R46L variants accumulate epicardial fat and have an increased left ventricular mass index, despite maintaining a normal left ventricular ejection fraction, underscoring the significance of PCSK9 in cardiomyocyte lipid uptake balance (Baragetti et al., 2017; Da Dalt et al., 2021). However, in another extensive nested case-control study, PCSK9 LOF carriers displayed left ventricular size, ejection fraction, and heart failure prevalence comparable to those in normal individuals (Trudsø et al., 2023).

In summary, PCSK9 is released from the myocardium, rather than from circulation, and actively preserves myocardial lipid homeostasis. The absence of PCSK9 within the myocardium leads to disruption of lipid metabolism, myocardial dysfunction, and potential heart failure. However, PCSK9 LOF carriers in the general population do not exhibit the same degree of myocardial damage as that observed in PCSK9 knockout animals and *in vitro* models. Possible explanations, which remain to be comprehensively examined, include the partial retention of lipid regulatory effects in low-expressing PCSK9 myocardium compared to knockout models or enhanced compensatory mechanisms in human cardiomyocytes against PCSK9 deficiency.

## 4 PCSK9 protects lipid metabolism in β-cells

### 4.1 β-cells and lipid toxicity

The American Diabetes Association characterizes T2DM as progressive insulin insufficiency coupled with insulin resistance (2, 2022). Central to T2DM pathology is the demise of islet β-cells, driven by factors like lipid toxicity, glucotoxicity, and amyloid formation (Stumvoll et al., 2005; Eizirik et al., 2020). Excessive cholesterol accumulation within islet cells fosters lipid toxicity, impeding insulin secretion, and inducing β-cell death (Perego et al., 2019; Tricò et al., 2022). The lipid buildup curtails ATP production by inhibiting glycolysis, depletes calcium stores that are necessary for insulin secretion, and alters insulin particle formation, all damaging the release process of β-cells (Cnop et al., 2002; Lu et al., 2011; Bogan et al., 2012). Additionally, the accumulation of cholesterol on mitochondrial membranes impairs mitochondrial function, accumulation on endoplasmic reticulum triggers endoplasmic reticulum stress, and accumulation on cytoplasmic membranes triggers apoptosis proteins, leading to β-cell death (Lu et al., 2011; Paul et al., 2016; Lytrivi et al., 2020). Notably, β-cell lipotoxicity is primarily driven by the accumulation of LDL-C, while HDL-C averts β-cell apoptosis (Rütti et al., 2009). In LDLR knockout mice, LDLR is essential for β-cell LDL-C uptake, mediating lipotoxicity (Kruit et al., 2010).

### 4.2 Deficiency of PCSK9 in β-cells and association with diabetes

PCSK9 is detectable in islets and regulates the abundance of LDLR on the surface of β-cells (Tchéoubi et al., 2022). While many studies suggest an autocrine function of β-cells, a paracrine role of PCSK9 from δ-cells impacting β-cells cannot be excluded (Mbikay et al., 2010; Da Dalt et al., 2019). In PCSK9 knockout mice, pancreatic islets displayed anomalous contours, inflammatory cell infiltration, and early β-cell apoptosis (Mbikay et al., 2010). High glucose levels and relative insulin deficiencies were evident in the blood (Mbikay et al., 2010). Subsequent investigations indicated that PCSK9 knockout boosted LDLR density on β-cell surfaces, with a large presence of lipid droplets and immature insulin secretion particles within cells, despite low plasma insulin levels (Mbikay et al., 2015; Da Dalt et al., 2019). These findings imply that PCSK9 deficiency-driven lipid accumulation does not affect insulin synthesis but does impair β-cell secretory function. Remarkably, in PCSK9 knockout models, females displayed relatively normal glucose disposal compared to glucose-intolerant males, who manifested impaired plasma glucose and glucose-stimulated insulin secretion (Mbikay et al., 2015; Roubtsova et al., 2015). Ovariectomies in females mirrored the islet damage observed in males, and estrogen treatment reversed this effect (Roubtsova et al., 2015). The ability of estrogen to protect against apoptosis through its interaction with estrogen receptors on β-cells suggests an islet-protective role in the absence of PCSK9 (Babiloni-Chust et al., 2022; Sharma and Prossnitz, 2022). Notably, β-cell lipotoxicity induced by PCSK9 deficiency was reversed in PCSK9 and LDLR double-knockout mice, implying that LDLR-based lipid uptake pathways underlie this damage (Da Dalt et al., 2019). Furthermore, liver-specific PCSK9 knockout prevented lipid accumulation and restored islet β-cell secretory function, suggesting that localized islet PCSK9 regulates LDLR degradation, rather than serum PCSK9 levels (Da Dalt et al., 2019). Conversely, pancreatic-specific PCSK9 knockout boosted LDLR on β-cell surfaces, despite normal serum PCSK9 levels, leading to insufficient insulin secretion (Marku et al., 2022). Surprisingly, PCSK9 deficiency appears to trigger a protective strategy against lipid accumulation in β-cells (Marku et al., 2022). Enhanced expression of ATP-binding cassette transporter A1 (ABCA1), ATP-binding cassette transporter G1, and liver X receptor has been noted in β-cells, suggesting heightened lipid efflux to combat lipid buildup (Brunham et al., 2007; Kruit et al., 2012). Moreover, proteins responsible for cholesterol esterification, including acetyl coenzyme A acetyltransferase 1, and sterol O-acyltransferase 1, are reportedly significantly upregulated (Da Dalt et al., 2019; Marku et al., 2022). As key players in cholesterol esterification, their increased levels aid cholesterol consumption (Chang et al., 2006).

The islet-protective effects of PCSK9 have also been reported in humans. A meta-analysis linking PCSK9 LOF variants and diabetes risk demonstrated the correlation between decreased LDL-C and increased risk of diabetes, with an odds ratio of 1.19 (95% confidence interval 1.02–1.38) for each 1 mmol/L decrease in LDL-C (Lotta et al., 2016). This trend is consistent with a higher diabetes risk in individuals harboring PCSK9 LOF variants with impaired fasting glucose at baseline, despite a reduced risk of CAD (Ference et al., 2016). In a Mendelian randomized study, PCSK9 LOF mutations causing low LDL-C levels were associated with elevated fasting glucose, body weight, and an increased risk of new-onset diabetes (Schmidt et al., 2017). Nonetheless, several studies have reported that PCSK9 LOF variants do not alter fasting blood glucose or insulin levels and are not linked to diabetes development (Bonnefond et al., 2015; Chikowore et al., 2019). This discrepancy could be explained by the observation that PCSK9 levels in humans are typically reduced by approximately 15% in LOF variants, which is significantly lower than that in PCSK9 knockout animal models. Hence, human PCSK9 deficiency effects might be compensated for more effectively (Humphries et al., 2009).

Notably, merely knocking out PCSK9 may not impair β-cell insulin secretion function, despite the observed substantial upregulation of LDLR on β-cell surfaces (Langhi et al., 2009). In a specific knockout mouse model focused on β-cells, no irregularities in diabetes-related markers were observed, and islet function remained unaffected (Peyot et al., 2021). However, mRNA levels of LDLR and 3-hydroxy-3-methylglutaryl-coenzyme A reductase decreased by 32% and 29%, respectively, indicating that β-cells might curb endogenous cholesterol synthesis to forestall excessive lipid buildup (Peyot et al., 2021). Examination of human β-cells cultured *in vitro* demonstrated that both secreted and exogenous PCSK9 could influence LDLR density, yet the absence of PCSK9 from either source did not disrupt insulin secretion (Ramin-Mangata et al., 2021). Notably, serum PCSK9 does not appear to degrade β-cell LDLR (Da Dalt et al., 2019; Marku et al., 2022).

To summarize, the impact of the lack of PCSK9 secreted by β-cells on islet function remains contentious. The reported reduction in LDLR degradation due to PCSK9 autocrine absence heightens lipid uptake by β-cells. Conversely, islet β-cells appear to exhibit a compensatory capacity against lipid accumulation. This manifests as diminished lipoprotein receptor synthesis, reduced *de novo* cholesterol synthesis, increased receptor excretion of surplus lipids, and augmented cholesterol esterification (Table 1). Furthermore, research has indicated that glucagon secreted by pancreatic α-cells can inhibit PCSK9 expression. Given that many diabetic patients have increased glucagon production and decreased insulin production, could this lead to β-cell lipotoxicity related to islet PCSK9 deficiency? These questions warrant further investigation (Folli et al., 2018; Spolitu et al., 2019). Insulin upregulates liver PCSK9 via the SREBP1-c pathway (Costet et al., 2006), indicating the possibility that insulin promotes autocrine PCSK9 expression as a mechanism for regulating β-cell lipid homeostasis.

**TABLE 1:** Effects of islet autocrine PCSK9 deficiency on islet β cell function and diabetes risk.

Authors	Study models	Lipoprotein receptors on β cells	Effects on islet	Diabetes risk	Ref.
Langhi, C. et al. 2009	PCSK9 knockout mice	LDLR protein↑	PCSK9 autocrine deficiency did not change the content and composition of cholesterol in islets	PCSK9 autocrine deficiency did not affect fasting glucose levels, insulin levels, or GSIS	Langhi et al. (2009)
Mbikay, M. et al. 2010	PCSK9 knockout mice	LDLR mRNA↑ LDLR protein↑	PCSK9 autocrine deficiency induced abnormal islet morphology, inflammatory cell infiltration, islet cells apoptosis, and less insulin in the islet	PCSK9 autocrine deficiency induced hyperglyce- mia, hypoinsulinemia, and glucose intolerance developed in mice	Mbikay et al. (2010)
Da Dalt, L. et al. 2019	PCSK9 knockout mice; liver-specific PCSK9 knockout mice	LDLR, ACAT1 mRNA↑ HMGCR mRNA↓ LDLR protein↑	PCSK9 autocrine deficiency resulted in enlarged islets, insulin accumulation in beta cells, and the accumulation of mitochondria -related lipid droplets	PCSK9 autocrine deficiency induced impaired glucose tolerance, without insulin resistance and hyperglycemia	Da Dalt et al. (2019)
Peyot, M. L. et al. 2021	PCSK9 knockout mice; pancreas -specific PCSK9 knockout mice	LDLR, HMGCR mRNA↓ LDLR protein↑	PCSK9 knockout mice had normal insulin levels in the islets. Pancreas-specific PCSK9 knockout mice had higher insulin content in islets due to active basal protein secretion	PCSK9-deficient mice showed normal glucose tolerance and insulin sensitivity despite in- creased basal insulin secretion	Peyot et al. (2021)
Ramin-Mangata, S. et al. 2021	Human pancreatic β cell	LDLR protein↑	Although PCSK9 affected LDLR concentration and LDL-C uptake in β cells, neither endoge- nous nor exogenous PCSK9 deficiency affected insulin secretion	PCSK9 autocrine deficiency did not affect GSIS	Ramin-Mangata et al. (2021)
Marku, A. et al. 2022	Pancreas-specific PCSK9 knockout mice	LDLR, ABCA1, ABCG1, LXR, SOAT1 protein↑	PCSK9 autocrine deficiency leads to increased lipid uptake by β cells and intracellular accumulation of cholesterol and insulin	Pancreas-specific PCSK9 knockout mice had normal circulating cholesterol levels but had glucose intolerance and hypoinsulinemia	Marku et al. (2022)
Bonnefond, A. et al. 2015	PCSK9 p.R46L genetic variant	—	—	PCSK9 LOF was not associated with glucose homeostasis (FPG, HbA1c, HOMA-IR), fasting insulin levels and diabetes incidence but was associated with elevated fasting glucose levels	Bonnefond et al. (2015)
Ference, B. A. et al. 2016	PCSK9 variants	—	—	PCSK9 LOF mutations increased the incidence of diabetes, but only in individuals with impaired fasting glucose and on an order of magnitude less than the protection of the cardiovascular system	Ference et al. (2016)
Lotta, L. A. et al. 2016	PCSK9 variants	—	—	A decrease in serum LDL-C concentration is associated with an increased risk of new-onset diabetes	Lotta et al. (2016)
Lotta, L. A. et al. 2016	PCSK9 variants	—	—	A decrease in serum LDL-C concentration is associated with an increased risk of new-onset diabetes	Kleinewietfeld and Hafler (2013)
Chikowore, T. et al. 2019	PCSK9 variants	—	—	PCSK9 LOF is associated with lower fasting blood glucose levels during adolescence	Chikowore et al. (2019)

## 5 Neuroprotective effect of PCSK9 in the brain

In rodent studies, no alteration in brain LDLR levels was observed following adenovirus-mediated overexpression of PCSK9 in the liver or by injection of recombinant PCSK9. These findings indicate that serum PCSK9 cannot breach the blood-brain barrier (Schmidt et al., 2008). This assertion is further supported by the substantial disparity between PCSK9 concentrations in cerebrospinal fluid and serum PCSK9 levels, along with the lack of circadian rhythm synchronization between the two (Chen et al., 2014). These observations collectively underscore the significance of brain-derived autocrine PCSK9, rather than hepatic PCSK9 within the brain.

The expression of PCSK9 in the brain exhibits regional specificity. During embryonic development of zebrafish, PCSK9 is highly expressed in the notochord, cerebral cortex, cerebellar granulosa cell precursors, and other neural-forming regions (Poirier et al., 2006). Prominent PCSK9 expression in the frontal cortex of mouse embryos has been described (Rousselet et al., 2011). In contrast, the adult mouse brain has significantly lower PCSK9 expression than its fetal counterpart, with a prevalence in regions of sustained neurogenesis, such as the outer granular layer and rostral extension of the cerebellar olfactory peduncle (Seidah et al., 2003). Collectively, these observations strongly hint at a role of PCSK9 in neurodevelopment. In zebrafish embryos injected with PCSK9 mRNA inhibitors, abnormal neurogenesis was observed 24 h post-fertilization, as evidenced by cerebellar neuron disarray, deletion of the parietal cap and posterior brain, and disappearance of the posterior midbrain boundary. The peak mortality rate was observed at 48–96 h post-fertilization (Poirier et al., 2006). During retinoic acid-induced differentiation of mouse embryonic pluripotent cells, PCSK9 mRNA levels peaked on the 2nd day. Simultaneously, SREBP2 mRNA and LDLR protein levels exhibited negligible changes, indicating that the influence of PCSK9 on neurogenesis was independent of LDLR (Poirier et al., 2006). Similarly, PCSK9 deficiency in adult mice did not affect LDLR levels within the olfactory bulb (Rousselet et al., 2011). In rodent models, PCSK9 levels were notably diminished in both neural centers and the placenta of fetal mice with neural tube defects (An et al., 2015). This suggests that PCSK9 plays a pivotal role in fetal neural development and serves as a potential biomarker for the diagnosis of prenatal neural tube defect (An et al., 2015). Notably, although PCSK9 is essential for brain survival in certain species, its importance does not seem to extend to mammals. Experiments with PCSK9 knockout mouse embryos showed that the integrity of the telencephalon tissue remained intact, suggesting that PCSK9 is not particularly critical for mouse brain development (Rousselet et al., 2011). Furthermore, there were no indications of disorders in the stratification of the cerebral cortex or cerebellar structures in adult mice lacking PCSK9 (Rashid et al., 2005).

A long-term study involving African American individuals with PCSK9 LOF variants found no link between prolonged exposure to low PCSK9 levels and neurocognitive impairment or cognitive decline (Mefford et al., 2018). Similarly, a randomized controlled study involving European participants revealed no significant differences in neurocognitive function, intelligence, memory, or brain gray or white matter volumes between PCSK9 LOF variant carriers and controls (Ghouse et al., 2022). Even in cases of complete PCSK9 LOF mutations, in which serum PCSK9 is undetectable, individuals exhibit normal survival and fertility (Zhao et al., 2006; Hooper et al., 2007). Considering the potential positive effect of PCSK9 on neurodevelopment, several studies have explored the association between PCSK9 monoclonal antibodies and neurocognitive diseases. Analysis of pooled data from 14 trials indicated no notable increase in overall adverse events, including neurological disorders, associated with the use of PCSK9 inhibitors (Robinson et al., 2017).

In summary, although PCSK9 deficiency negatively affects neurodevelopment in experimental models, this effect seems to be less severe in humans. This can be explained from three perspectives. First, the complexity of the lipid metabolism pathway in the mammalian brain compared with that in fish may enable compensatory mechanisms that mitigate the effects of PCSK9 deficiency. Second, owing to the challenge of lipoprotein penetration through the blood-brain barrier, the brain predominantly relies on neurons and glial cells for *de novo* cholesterol synthesis, which maintains the stability of the cholesterol pool (Orth and Bellosta, 2012). Third, the passage of PCSK9 monoclonal antibodies into the brain via the blood-brain barrier is challenging.

## 6 PCSK9 regulates sodium reabsorption in the kidney

The Epithelial Sodium Channel (ENaC) non-voltage-gated ion channel protein is widely expressed in the kidneys, lungs, distal colon, sebaceous glands, eccrine glands, and other tissues, facilitating the transcellular absorption of sodium ions (Hanukoglu et al., 2017). In the kidney, ENaC resides in the luminal membrane of the distal tubules and collecting ducts of the distal nephrons. Its activity is influenced by salt intake and mineralocorticoid secretion (Zhang et al., 2022). Given its role in sodium absorption and blood volume maintenance, gain-of-function and deletion mutations in ENaC can lead to severe salt-sensitive hypertension and hypotension, respectively (Bonny and Hummler, 2000; Furuhashi et al., 2005). PCSK9 is notably abundant in the distal renal collecting duct, making it the second-largest source of PCSK9 after the liver (Seidah et al., 2003; Liu and Vaziri, 2014). Unlike LDLR endocytosis, PCSK9 orchestrates ENaC degradation via the proteasomal pathway. This action curtails the intracellular ENaC pool, suppresses ENaC exocytosis, and reduces ENaC density on the cell membrane surfaces (Sharotri et al., 2012). In a PCSK9 knockout mouse model, renal ENaC expression increased by nearly one-third, but blood pressure and sodium homeostasis remained unaffected (Berger et al., 2015). Post-amiloride ENaC inhibition and urinary sodium excretion increased comparably in wild-type and PCSK9 knockout mice, further underscoring the lack of physiological impact of PCSK9 deficiency on ENaC function (Berger et al., 2015).

Consequently, there is a discordance in the link between PCSK9 deficiency and blood pressure in humans. Among Caucasians, p.R46L variants do not increase the risk of hypertension compared to controls (Zhao et al., 2006). In an exploration of the association between PCSK9 genetic variants and blood pressure in African Americans, the PCSK9 variant was found to have a modest influence on diastolic blood pressure (Tran et al., 2015). However, in a male population of Asian descent, the PCSK9 p.R46L group demonstrated markedly higher blood pressure than non-carriers (Jeenduang et al., 2015). In one unique case, an individual bearing a PCSK9 LOF mutation barely expressed PCSK9 and did not manifest hypertension (Cariou et al., 2009). Furthermore, examination of patients with hypertension showed no correlation between blood pressure and serum PCSK9 levels (Yang et al., 2016).

In summary, there are disparities in research findings regarding the link between PCSK9 deficiency and salt-sensitive hypertension among Asian and African American groups. Thus, there may be racial disparities in the effects of PCSK9 deficiency on blood pressure. However, due to the scarcity of research data, the extent to which renal PCSK9 deficiency influences blood pressure regulation remains unclear. The establishment of additional animal models, such as specific renal collecting duct PCSK9 knockout models, is imperative to more precisely determine the regulatory effects of PCSK9 on ENaC and blood pressure.

## 7 PCSK9 is involved in intestinal lipid absorption

The efficient excretion of cholesterol through the intestine is important for maintaining optimal plasma cholesterol levels. In addition to the conventional hepatobiliary route, recent studies have revealed a novel mechanism termed transintestinal cholesterol efflux (TICE), which is particularly active in the proximal intestine (van der Velde et al., 2007). The efficacy of TICE hinges on multiple factors. One factor is the role of ApoB-48 in basolateral membrane binding to chylomicrons, facilitating their reuptake via LDLR presentation. PCSK9 influences this reuptake by modulating LDLR density (Le May et al., 2009). Another factor is the complementary cooperation between the ATP-binding cassette transporter G5/G8 and ABCB1 proteins in the apical intestinal epithelial membrane, which orchestrates cholesterol transport from the lumen to the interior (Hui et al., 2008; Le May et al., 2013; Dugardin et al., 2017). In rodents, approximately one-third of the total fecal cholesterol discharge occurs through TICE, doubling the amount via bile pathways, underscoring the dominant role of TICE in cholesterol elimination (van der Veen et al., 2009). TICE can be activated by food and medications that include phytosterols, bile acids, fasting, liver X receptor agonists, peroxisome proliferator-activated receptor agonists, ezetimibe, and statins (Tanaka and Kamisako, 2021; Garçon et al., 2022). In humans, TICE is also an important component of the body’s reverse cholesterol transport (RCT) process. It is estimated that 35% of fecal cholesterol is produced through the TICE pathway (Garçon et al., 2022). In humans, TICE is also inducible, clinical studies have found that treatment with 10 mg/day of the lipid-lowering drug ezetimibe for 4 weeks can enhance TICE by fourfold (Jakulj et al., 2016). These findings has spurred research aimed at lipid reduction. Compared with hepatobiliary stimulation, TICE activation has fewer adverse effects, making it a promising avenue for further investigation (Garçon et al., 2022).

Within the gastrointestinal tract of rodents, abundant PCSK9 and LDLR expression spans the small intestine to the colon. The PCSK9 and LDLR levels are harmoniously distributed along the cephalocaudal axis of the intestine (Le May et al., 2009). Immunofluorescence staining has revealed that PCSK9 primarily resides within the intestinal epithelium, including goblet cells and enterocytes, and is prominently situated on both the basolateral and apical facets (Le May et al., 2009). However, whether PCSK9 produced by the intestinal cells can enter the bloodstream remains debatable. In the early stages of differentiation of the Caco-2 colon cancer cell line, PCSK9 secretion from the basolateral compartment was observed (Levy et al., 2013; Moreau et al., 2021). However, after the differentiation and maturation of Caco-2 cells, PCSK9 secretion reportedly became negligible (Moreau et al., 2021). Despite significant PCSK9 protein detection in human and rodent intestinal tissues, *in vitro* cultivation of these intestinal tissues did not reveal PCSK9 secretion, suggesting a predominantly autocrine role for PCSK9 expressed by intestinal cells (Moreau et al., 2021).

PCSK9 knockout upregulates TICE *in vivo* and *in vitro* (Le May et al., 2013). Studies involving rodents have indicated that increased chylomicron clearance in PCSK9 knockout mice leads to a significant postprandial reduction in triglyceride (Le May et al., 2009). However, while PCSK9 knockout results in decreased ApoB secretion in intestinal cells, which logically leads to a decrease in the number of triglyceride-rich chylomicrons, there is a compensatory increase in the volume of these structures (Le May et al., 2009). Subsequent investigations revealed that PCSK9 knockout can trigger a notable increase in intestinal LDLR levels (Le May et al., 2009). Considering that the clearance of chylomicrons remnants relies largely on the LDLR-ApoB pathway, it is plausible to speculate that a deficiency in intestinal PCSK9 promotes the reuptake of chylomicrons by increasing intestinal LDLR levels, thereby reducing postprandial plasma LDL-C and triglyceride levels (Le May et al., 2009). These findings pave the way for potential lipid regulation treatments centered on the intestinal PCSK9/LDLR axis. Dietary therapy involving the acute intragastric administration of plant sterols in rodents resulted in a five-fold increase in intestinal LDLR expression, greatly enhancing TICE (De Smet et al., 2015). Similarly, exercise training in rodents leads to elevated LDLR levels in the basolateral membrane of the intestinal canal, further boosting TICE (Farahnak et al., 2018). Interventions involving a rodent diet and exercise have also yielded interesting results, such as significant upregulation of intestinal PCSK9 expression. This could be partially explained by the upregulation of SREBP2 expression in the intestine, whereas hepatic PCSK9 expression was inhibited. However, this phenomenon warrants further investigation to provide a more comprehensive explanation (De Smet et al., 2015; Farahnak et al., 2018).

Although preliminary studies in animal and *in vitro* models have revealed the impact of PCSK9 on TICE, relevant clinical research on the impact of PCSK9 on TICE in humans is still lacking. Future clinical research is needed to observe changes in TICE among individuals with PCSK9 LOF mutations and patients using PCSK9 inhibitors.

## 8 Protective effect of PCSK9 in other tissues

### 8.1 PCSK9 protects lipid metabolism in seminiferous tubules

The testes are partitioned into two cellular compartments by the blood-testosterone barrier: the interstitium, which is primarily responsible for lipid metabolism and androgen synthesis, and the seminiferous tubule, which is responsible for germ cell growth and development. Within seminiferous tubules, LDLR-mediated lipid transport plays a crucial role in maintaining high lipid levels and providing essential nutrients necessary for spermatogonial division and differentiation (Schenk and Hoeger, 2010). Although PCSK9 mRNA has been detected in the testis, its length (2.2 kb) differs from that in other tissues (2.8 kb) (Seidah et al., 2003). PCSK9 has been identified in the adipose tissue of the epididymis, interstitial tissue of the testis, sperm tubules, in rodents (Pelletier et al., 2022). Recent research has highlighted the role of PCSK9 in regulating lipid metabolism to maintain seminiferous tubule function. In PCSK9 knockout mice, cholesterol accumulation and immune cell infiltration were observed in the seminiferous tubules, accompanied by increased LDLR levels and the presence of the inflammatory factor interleukin-17 (Pelletier et al., 2022). This cytokine, secreted mainly by highly infiltrating γδT cells in the testis, has been linked to macrophage polarization and autoimmune responses in experimental orchitis models (Park et al., 2005; Kleinewietfeld and Hafler, 2013; Wilharm et al., 2021). Excessive cholesterol accumulation promotes expression of this interleukin and creates an inflammatory environment that contributes to seminiferous tubule dysfunction (Wang et al., 2015; Varshney et al., 2016; Kim et al., 2019).

### 8.2 PCSK9 prevents abnormal distribution of adipose tissue and local inflammation

PCSK9 is expressed in visceral adipose tissue and is regulated by natriuretic peptides and insulin (Bordicchia et al., 2019). Within adipose tissue, the primary influences of PCSK9 appear to be on CD36 and VLDLR, involving the intake and accumulation of FAs, rather than on LDLR (Roubtsova et al., 2011; Christiaens et al., 2012; Demers et al., 2015). Among these receptors, CD36 governs the differentiation of pre-adipocytes into mature adipocytes, and the absence of CD36 significantly diminishes the subcutaneous and gonadal fat content in mice (Christiaens et al., 2012). *In vitro* experiments involving adipocytes have revealed a three-fold increase in CD36 expression and uptake of oxidized LDL following PCSK9 knockout. This genetic alteration leads to ectopic fat accumulation in the visceral organs of mice (Baragetti et al., 2017).

In individuals carrying PCSK9 LOF variants, heightened visceral fat thickness, including central obesity, liver steatosis, and epicardial fat, has been detected. These changes appear to be tied to adipocyte hypertrophy and inflammatory responses (Baragetti et al., 2017; Hay et al., 2022). Given these findings, it is reasonable to infer that the presence of PCSK9 in adipose tissue contributes to the balanced distribution of body fat through the regulation of adipocyte metabolism.

Although LDLR is not typically regarded as the primary pathway for lipid uptake in adipose tissue, particularly in white adipose tissue, including epicardial adipose tissue, low levels of PCSK9 can trigger upregulated LDLR expression within adipocytes (Dozio et al., 2020), consequently prompting additional uptake of LDL-C (Dozio et al., 2020). The accumulation of excess LDL-C in adipocytes can initiate localized inflammation and insulin resistance by activating the NOD-like receptor thermal protein domain-associated protein 3 (NLRP3) inflammatory corpuscles. These activated inflammatory corpuscles induce mitochondrial dysfunction and insulin resistance by activating macrophages infiltrated within adipose tissue (Dozio et al., 2020; Javaid et al., 2023). Notably, among obese individuals with low serum PCSK9 concentrations, the surface expression of LDLR and CD36 increased by 81% and 36%, respectively, on white adipose tissue cells (Cyr et al., 2021). This led to a corresponding elevation in the activation level of NLRP3 inflammatory corpuscles and increased susceptibility to diabetes mellitus, surpassing that observed in other subjects (Cyr et al., 2021). The fact that PCSK9 exhibits an anti-inflammatory effect within adipose tissue is intriguing, given that it is typically considered a pro-inflammatory factor (Ding et al., 2020; Punch et al., 2022). This implies that PCSK9 has dual functions in inflammatory reactions, exerting both anti- and pro-inflammatory effects. This novel hypothesis requires validation in various tissue types other than adipose tissue.

### 8.3 PCSK9 deficiency aggravates apical periodontitis

PCSK9 deficiency significantly influences apical periodontitis. Gram-negative bacteria mainly drive this chronic inflammatory ailment, particularly *Porphyromonas gingivalis*, which infiltrates periodontal support tissue (Ye et al., 2023). The expression of PCSK9 was increased in a mouse model of apical periodontitis induced by *P. gingivalis* and in the gingival tissues of patients with periodontitis (Sun et al., 2018). Although PCSK9 can promote the release of pro-inflammatory cytokines and exacerbates the inflammatory response, it can also facilitate the osteogenic differentiation of periodontal ligament stem cells (Sun et al., 2018). However, in cases of PCSK9 deficiency, LDLR expression within periodontal tissue increases, intensifying the differentiation of bone marrow macrophages to osteoclasts and amplifying cementum loss (Huang et al., 2022). This cascade of events hinges on LDLR dependence, as evidenced by experiments involving LDLR knockout, which reportedly arrested the worsened progression of apical periodontitis in a state of PCSK9 deficiency (Huang et al., 2022).

## 9 Advancements in the clinical benefits and safety of PCSK9 inhibition therapy

### 9.1 PCSK9 and vasculature

Within the vasculature, PCSK9 serves as a pivotal regulator of LDL-C levels and acts as a driver of atherosclerosis and subsequent atherosclerotic cardiovascular diseases (ASCVD) (Boutari et al., 2023). This effect is manifest through the promotion of chronic vascular inflammation, formation of atherosclerotic plaques, and initiation of thrombosis (Boutari et al., 2023; Hummelgaard et al., 2023). In macrophages, the secretion of PCSK9 is triggered by oxidized LDL, leading to macrophage polarization via the Toll-like receptor 4/nuclear factor kappa B signaling pathway (Wang et al., 2022). PCSK9 is also secreted by vascular smooth muscle cells, which exhibit enhanced proliferation, migration, and foam cell formation induced by oxidized LDL, thus aggravating atherosclerosis (Liu et al., 2023). The influence of PCSK9 extends to platelets; PCSK9 secretion stimulates platelet activation, intensifies platelet-dependent thrombosis, and fosters thrombotic inflammatory reactions (Petersen-Uribe et al., 2021). In clinical practice, a noteworthy correlation has been established between the serum concentration of PCSK9 and the presence and proportion of atherosclerotic necrotic core tissues, as demonstrated by intramural ultrasound virtual histological imaging (Cheng et al., 2016). The focus of recent research has shifted to the impact of PCSK9 inhibition therapy on coronary plaque (Nicholls et al., 2016). In a double-blind randomized controlled trial involving patients with AMI, serial multimodal intracoronary imaging was performed (Räber et al., 2022). The percent atheroma volume in non-infarct related coronary arteries showed a more significant reduction in patients treated with PCSK9 monoclonal antibodies in combination with statins for 52 weeks compared to those treated with statins alone (−2.13% vs. −1.21%) (Räber et al., 2022). Furthermore, in an open-label, single-arm clinical trial involving patients with familial hypercholesterolemia but without clinical ASCVD, 78 weeks of PCSK9 monoclonal antibody alirocumab treatment led to a decrease in the coronary plaque burden from 34.6% to 30.4% (Pérez de Isla et al., 2023). Notably, there are changes in the characteristics of coronary artery plaques, with an increase in the proportion of calcified and mainly fibrous plaques, along with a decrease in necrotic and fibrous fatty plaques (Pérez de Isla et al., 2023). The collective findings indicate the benefits of PCSK9 inhibition therapy on the volume, composition, and phenotype of coronary plaque (Räber et al., 2022; Pérez de Isla et al., 2023).

PCSK9 LOF variants associated with congenital PCSK9 deficiency reportedly exhibited a 14% reduction in plasma LDL-C levels and a 21% decrease in TG levels compared with non-carriers (Ooi et al., 2017). A comprehensive meta-analysis of nine studies on PCSK9 LOF variants further revealed variations in plasma LDL-C levels among black and white populations, with reductions of 35 and 13 mg/dL, respectively. Importantly, both groups exhibited a lower risk of CAD than non-carriers (Kent et al., 2017). Thus, PCSK9 LOF mutations provide substantial vascular protection in clinical settings.

### 9.2 PCSK9 inhibition and clinical cardiovascular benefits

PCSK9 inhibitors have promising potential in the treatment of ASCVD (Hummelgaard et al., 2023). Beyond well-established monoclonal antibodies such as evolocumab and alirocumab, which target the PCSK9 protein, and small interfering RNA (siRNA) therapies, such as inclisiran targeting PCSK9 mRNA, various innovative approaches, including gene editing, vaccines, and peptides, have been explored (Hummelgaard et al., 2023). In a randomized, double-blind, prospective controlled trial involving patients with ASCVD, the incidence of the primary endpoint (9.8% vs. 11.3%) and critical secondary endpoint (5.9% vs. 7.4%) after 48 weeks of treatment with a PCSK9 monoclonal antibody was significantly lower than that in the control group (Sabatine et al., 2017). Similarly, a comprehensive analysis of multiple Phase III trials found that after 90 days of treatment with PCSK9 siRNA, the incidence of composite major adverse cardiovascular events (MACE) was notably reduced (7.1% vs. 9.4%) compared with the placebo group (Ray et al., 2023a). Patients who undergo percutaneous coronary intervention (PCI) usually face a heightened risk of MACE (Furtado et al., 2022). In a randomized controlled study with a median follow-up period of 2.2 years, patients with a history of PCI were treated with PCSK9 monoclonal antibodies, resulting in a significant reduction in the incidence of MACE (11.2% vs. 13.2%) and risk of vascular remodeling (7.2% vs. 9.3%), as reported previously (Furtado et al., 2022).

Furthermore, beyond their primary and secondary preventive applications in ASCVD, the clinical use of PCSK9 inhibitors has been increasing. Recent research has shifted its focus toward the feasibility of applying PCSK9 inhibition therapy to patients with acute coronary syndrome (ACS) as soon as possible, as both serum and ischemic myocardial PCSK9 levels surge rapidly during ACS, potentially contributing to acute inflammatory reactions (Cariou et al., 2017; Ding et al., 2018; Ferri et al., 2022). In a placebo-controlled trial in patients with AMI, the PCSK9 monoclonal antibody treatment group was treated with alirocumab within 24 h of emergency PCI (Räber et al., 2022). After 52 weeks, the alirocumab treatment group exhibited a significantly lower incidence of adverse events (70.7% vs. 72.8%) and coronary revascularization (8.2% vs. 18.5%) than the placebo group (Räber et al., 2022). In another prospective randomized controlled study among extremely high-risk ACS patients, patients were randomly assigned to the evolocumab group or placebo group at a ratio of 1:1, and the first medication was administered within 48 h after PCI (Hao et al., 2022). During the 3 months follow-up period, MACE incidences were significantly lower in the evolocumab group than in the placebo group (8.82% vs. 24.59%). In summary, PCSK9 inhibition therapy has significant cardiovascular benefits in patients with ACSVD (Hao et al., 2022).

### 9.3 Advances in clinical studies on the safety of PCSK9 inhibition

While PCSK9 inhibition therapy has become increasingly pivotal in lipid reduction and cardiovascular event management, concerns regarding its safety have arisen given the vital role of PCSK9 in overall physiological metabolism and organ function. In the ODYSSEY open-label extension study, spanning an average observation period of 2.5 years, no significant increase in sudden adverse events was observed in patients with familial hypercholesterolemia undergoing PCSK9 monoclonal antibody treatment (Farnier et al., 2018). Similarly, a more extended FOURIER-OLE study with a median follow-up time of 5 years concluded that PCSK9 inhibitor use did not significantly increase the incidence of serious adverse events, including neurocognitive impairment or new-onset diabetes mellitus in patients with ASCVD (ODonoghue et al., 2022). Additionally, an open-label extension study assessing the safety of inclisiran reported a mere 1% incidence of serious safety adverse events after 4 years of drug intervention, equivalent to that in the control group (Ray et al., 2023b). Recognizing the potential negative effects of statins on neurocognitive function, multiple studies have investigated the relationship between PCSK9 inhibitors and neurocognitive function (Shahid et al., 2022). A systematic review of seven studies found no association between the use of PCSK9 monoclonal antibodies and neurocognitive events (Shahid et al., 2022). While statins can slightly elevate the risk of new-onset diabetes mellitus, mainly by reducing sensitivity to blood sugar fluctuations via inhibiting islet β-cell glucose transporter-2 and insulin receptors in tissues, current clinical evidence suggests that PCSK9 monoclonal antibodies are sufficiently safe (McNamara et al., 2009; Gotoh and Negishi, 2015). Although PCSK9 inhibition therapy may lead to a slight increase in hyperglycemia, this increase is not significant enough to induce new-onset diabetes mellitus (Guo Y. et al., 2021; Carugo et al., 2022). Furthermore, there remains a notable gap in clinical research regarding the potential adverse effects of PCSK9 silencing in extrahepatic organs. The clinical trials mentioned in this chapter are summarized in Table 2.

**TABLE 2:** Clinical trials.

Trail	Number	Duration	Target population	Intervention	Primary endpoints	Secondary endpoints	Significant outcomes	Reference
ATHEROREMO-IVUS study	581	—	Patients who underwent coronary angiography for acute coronary syndrome (ACS) or stable angina	—	—	—	Circulating PCSK9 levels are positively correlated with the volume of the plaque necrotic core tissue	Cheng et al. (2016)
GLAGOV randomized clinical trial	968	76 weeks	Patients with coronary artery disease taking statins	Evolocumab 420 mg monthly	The nominal change in percent atheroma volume	Percentage of patients demonstrating plaque regression	Evolocumab significantly reduces the volume of atherosclerotic plaques	Nicholls et al. (2016)
PACMAN-AMI randomized clinical trial	300	52 weeks	Patients undergoing percutaneous coronary intervention for acute myocardial infarction	Alirocumab 150 mg biweekly	The change in percent atheroma volume of non-infarct-related coronary arteries	Changes in maximum lipid core burden index and minimal fibrous cap thickness	Alirocumab significantly improves the regression of plaques in non-infarct-related coronary arteries	Räber et al. (2022)
ARCHITECT study	104	78 weeks	Patients with familial hypercholesterolemia without clinical ASCVD	Alirocumab 150 mg biweekly	Coronary plaque burden	Atherosclerotic Plaque Volume, Architecture and Composition	Alirocumab reduces plaque burden, increases the volume of calcified/fibrotic plaques, and reduces necrotic tissue volume	Pérez de Isla et al. (2023)
FOURIER trial	27,564	2.2 years	Patients with ASCVD and LDL-C ≥1.8 mmol/L	Evolocumab 420 mg monthly/140 mg biweekly	Composite of cardiovascular death, myocardial infarction (MI), stroke, hospitalization for unstable angina, or coronary revascularization	Composite of cardiovascular death, myocardial infarction, or stroke.	Evolocumab significantly lowers the risk of primary and secondary endpoint events	Sabatine et al. (2017)
ORION-9, -10 and −11 study	3,655	18 months	Patients with heterozygous familial hypercholesterolaemia, ASCVD, or ASCVD risk equivalent on maximally tolerated statin-therapy	Inclisiran 284 mg On days 1, 90, and 6-monthly	Non-adjudicated CV death, cardiac arrest, non-fatal MI, and fatal and non-fatal stroke	Total fatal and non-fatal MI, and stroke	Inclisiran significantly reduced composite MACE, but not fatal/non-fatal MIs or fatal/non-fatal stroke	Ray et al. (2023a)
PC-SCA-9 prospective study	174	—	Patients hospitalized for ACS	—	—	—	Serum PCSK9 levels are positively associated with severity of coronary artery lesions in ACS	Cariou et al. (2017)
ODYSSEY program	985	3.2 years	Patients diagnosed with heterozygous familial hypercholesterolemia	Alirocumab 75/150 mg biweekly	The long-term safety of alirocumab (treatment-emergent adverse events, laboratory data, and vital signs)	Efficacy of alirocumab on lipid parameters and the long-term immunogenicity of alirocumab	No long-term safety issues were observed with alirocumab	Farnier et al. (2018)
ORION-3 trial	382	4 years	Patients with prevalent ASCVD or high-risk primary prevention and elevated LDL cholesterol despite maximally tolerated statins or other LDL-lowering treatments	Inclisiran 300 mg 6-monthly	The percentage change in LDL-C	Changes in serum LDL-C and PCSK9 levels	The 4-year averaged mean reduction of LDL-C cholesterol was 44.2%, with reductions in PCSK9 ranging from 62·2% to 77·8%.	Ray et al. (2023b)
ORION-1 trial	501	240 days	Patients at high risk for cardiovascular disease who had elevated LDL cholesterol levels	Inclisiran 100, 200, or 300 mg at days 1 and 90	The change from baseline in LDL cholesterol level	Adverse event incidence rate	Patients who received inclisiran had dose-dependent reductions in PCSK9 and LDL--C levels	Liu et al. (2019)

The collective findings suggest that PCSK9 deficiency in clinical practice may not carry the same degree of harm as that observed in experimental models. However, in individuals with PCSK9 loss-of-function mutations and patients using PCSK9 inhibitors, PCSK9 levels are only partially reduced, far from the extent of PCSK9 knockout observed in experimental animal and *in vitro* models. Therefore, the potential negative consequences of a PCSK9 deficiency should not be ignored. Moreover, current research on the safety of PCSK9 inhibitors is limited by relatively short observation periods, making it challenging to ensure the long-term safety of continuous PCSK9 inhibition therapy, especially in high-risk patients who may require lifelong treatment. Extensive prospective studies are needed to ascertain whether such therapies can harm extrahepatic tissues. Currently, there are some shortcomings in the research on the safety of PCSK9 inhibitors, notably in proving their potential benefits or risks across different patient groups, particularly in high-risk patients with specific conditions. Additionally, when patients were administered PCSK9 inhibitors, there were substantial individual variations in PCSK9 and LDL-C levels. To assess whether PCSK9 inhibition poses a risk to extrahepatic organs, it is crucial to closely monitor individuals with significantly reduced levels of PCSK9 and LDL-C levels when using PCSK9 inhibitors. Monoclonal antibodies are the most widely used PCSK9 inhibitors in clinical practice. While such antibodies primarily counteract circulating PCSK9, it is essential to consider their potential impact on PCSK9 levels in extrahepatic tissues. The emergence of PCSK9 gene silencing therapies has further emphasized this concern. In contrast to monoclonal antibodies, PCSK9 siRNA and PCSK9 gene editing techniques that do not target the liver are more likely to interfere with PCSK9 synthesis in extrahepatic tissues.

## 10 Discussion

To date, despite the vigorous development of PCSK9 inhibitors, the understanding of the diverse physiological functions of PCSK9 in extrahepatic tissues remains incomplete. The lack of PCSK9 in mouse myocardium has been shown to affect myocardial contractility. However, these results are limited to animal knockout models. The impact of clinical use of PCSK9 inhibitors on myocardial contractility requires long-term clinical observation and rigorous clinical studies.

The relationship between PCSK9 and diabetes is complex. A deficiency of autocrine PCSK9 in the islets may impair β-cell function, leading to diabetes (Da Dalt et al., 2019). However, serum PCSK9 levels are generally elevated in diabetic patients (Ibarretxe et al., 2016). This suggests that the roles of islet-derived PCSK9 and liver-derived PCSK9 in diabetes may differ, necessitating more research to uncover the underlying mechanisms.

Existing evidence indicates that the function of PCSK9 in the brain may be independent of LDLR (Poirier et al., 2006). Future research should clarify whether the neuroprotective effects of PCSK9 depend on lipoprotein receptors other than LDLR or on specific non-lipid-related effects, and further elucidate the molecular mechanisms involved. Additionally, when exploring the effects of PCSK9 on the brain, special attention should be paid to differences in research conclusions due to species variations in experimental animals.

Current studies suggest that PCSK9 deficiency does not significantly affect blood pressure (Berger et al., 2015). However, given that PCSK9 does regulate renal ENaC, there is a clinical need to gather long-term follow-up data on the blood pressure of patients using PCSK9 inhibitors. Research on the impact of PCSK9 on TICE in the intestine is limited to laboratory findings. Clinical studies are needed to verify whether PCSK9 inhibitors can significantly stimulate TICE, similar to ezetimibe (Jakulj et al., 2016).

Generally, future research should aim to construct more tissue-specific PCSK9 knockout animal models and conduct more clinical studies, preclinical experiments, and interdisciplinary collaborations to better understand the roles of PCSK9 in extrahepatic tissues.

In human, studies indicate that, on average, the plasma PCSK9 level in PCSK9 LOF mutants is only reduced by 15%–20% compared to the normal population, with significant variability among individuals (Humphries et al., 2009; Lakoski et al., 2009; Wanneh et al., 2017). Future research should focus on comparing PCSK9-deficient mutant populations exhibiting the lowest PCSK9 expression with the normal population to ascertain any potential link between PCSK9 deficiency and associated diseases. Furthermore, clinical usage of PCSK9 inhibitors has not demonstrated an increased risk of serious safety adverse events, such as neurocognitive impairment or NODM. However, it is crucial not to overlook the substantial reduction of over 90% in serum PCSK9 levels observed within hours of clinical application of PCSK9 monoclonal antibodies, and this effect can last for 15 days (Gibbs et al., 2017; Liu et al., 2019). Inclisiran, another PCSK9 inhibitor, reduces serum PCSK9 levels by over 70% within 30 days and maintains this reduction for 180 days (Ray et al., 2017; Ray et al., 2019; Ray et al., 2023b). According to the frequency of use of these drugs, patients will be in a state of low PCSK9 level for a long time. Moreover, studies demonstrate that CRISPR base editing of PCSK9 can remarkably reduce PCSK9 expression in nonhuman primates by 90% (Musunuru et al., 2021). These findings highlight a more significant reduction of PCSK9 level in individuals using PCSK9 inhibitors long-term compared to PCSK9 LOF mutants. Consequently, the potential consequences of prolonged PCSK9 deficiency may be more severe in PCSK9 inhibitor users. It remains imperative to conduct comprehensive, long-term clinical monitoring to assess the safety and efficacy of PCSK9 inhibition therapy in different patient populations, especially those at high risk for extrahepatic tissue-related complications. In conclusion, while the clinical significance of PCSK9 circulation and localized inhibition is apparent, our understanding of the role(s) of PCSK9 in extrahepatic tissue remains limited. Circulating PCSK9 originates from the liver and primarily acts on the liver. All the effects of extrahepatic PCSK9 are autocrine and generally do not increase circulating PCSK9 levels. This may suggest that PCSK9 could have both intracellular and extracellular effects on these tissues. Although existing data show that the possibility of serious safety problems due to the application of PCSK9 inhibitors is low, long-term follow-up of their possible negative effects cannot be ignored when PCSK9 inhibitors are used clinically. This is the first review to delve into the pathophysiological interplay between PCSK9 deficiency and diverse extrahepatic tissue diseases. We hope that this review helps galvanize future research efforts toward unraveling protective contributions of PCSK9 in extrahepatic tissue.
